# Antiretroviral Therapy Dampens Mucosal CD4^+^ T Lamina Propria Lymphocytes Immune Activation in Long-Term Treated People Living with HIV-1

**DOI:** 10.3390/microorganisms9081624

**Published:** 2021-07-30

**Authors:** Alessandro Lazzaro, Giuseppe Pietro Innocenti, Letizia Santinelli, Claudia Pinacchio, Gabriella De Girolamo, Paolo Vassalini, Gianfranco Fanello, Claudio Maria Mastroianni, Giancarlo Ceccarelli, Gabriella d’Ettorre

**Affiliations:** 1 Department of Public Health and Infectious Diseases, Sapienza University of Rome, Policlinico Umberto I of Rome, 00185 Roma, Italy; letizia.santinelli@uniroma1.it (L.S.); claudia.pinacchio@uniroma1.it (C.P.); gabriella.degirolamo@uniroma1.it (G.D.G.); paolo.vassalini@uniroma1.it (P.V.); claudio.mastroianni@uniroma1.it (C.M.M.); giancarlo.ceccarelli@uniroma1.it (G.C.); gabriella.dettorre@uniroma1.it (G.d.); 2 Department of Emergency Surgery-Emergency Endoscopic Unit, Sapienza University of Rome, Policlinico Umberto I, 00185 Roma, Italy; gianfranco.fanello@endoroma.it

**Keywords:** HIV-1, antiretroviral therapy, ART, GALT, immune activation, Th1, Th17, mucosal immunology, microbiome

## Abstract

HIV infection is characterized by a severe deterioration of an immune cell-mediated response due to a progressive loss of CD4^+^ T cells from gastrointestinal tract, with a preferential loss of IL-17 producing Th cells (Th17), a specific CD4^+^ T cells subset specialized in maintaining mucosal integrity and antimicrobial inflammatory responses. To address the effectiveness of antiretroviral therapy (ART) in reducing chronic immunological dysfunction and immune activation of intestinal mucosa, we conducted a cross-sectional observational study comparing total IFN-γ-expressing (Th1) and IL-17-expressing (Th17) frequencies of CD4^+^ T lamina propria lymphocytes (LPLs) and their immune activation status between 11 male ART-naïve and 11 male long-term ART-treated people living with HIV-1 (PLWH) who underwent colonoscopy and retrograde ileoscopy for biopsies collection. Flow cytometry for surface and intracellular staining was performed. Long-term ART-treated PLWH showed lower levels of CD38^+^ and/or HLA-DR^+^ LPLs compared to ART-naïve PLWH. Frequencies of Th1 and Th17 LPLs did not differ between the two groups. Despite ART failing to restore the Th1 and Th17 levels within the gut mucosa, it is effective in increasing overall CD4^+^ T LPLs frequencies and reducing mucosal immune activation.

## 1. Introduction

A progressive loss of CD4^+^ T lymphocytes from blood, lymphoid organs and mucosal tissues is the hallmark of Human Immunodeficiency Virus 1 (HIV-1) infection. Such continuous depletion of CD4^+^ T lymphocytes leads to the acquired immune deficiency syndrome (AIDS) if specific antiretroviral therapy is not addressed promptly [1]. The gastrointestinal compartment dramatically suffers the lymphopenic effect of HIV-1 infection and plays a major role in the pathogenesis of HIV-1 infection itself [2]. Indeed, the gut hosts the gut-associated lymphoid tissue (GALT) and is believed to contain the large majority of the CD4^+^ T lymphocytes in the human body [3].

Within such scenario, a pivotal role is exerted by a specific CD4^+^ T lymphocytes subset producing IL-17 (Th17) [4], which are CD4+ T cells specialized in helping to maintain mucosal integrity and antimicrobial inflammatory responses [5]. The kinetic and amount of the Th17 loss occurring in the GALT soon after HIV-1 infection has been broadly reviewed [2,4] and nowadays it is clear that the gastrointestinal catastrophe established by HIV-1 is inversely related to the Th17 frequencies within the intestinal mucosa [6], and the latter is directly related to disease progression. Indeed, the quick yet gradual Th17 infection and depletion within the GALT lead to substantial alteration of the gut barrier competence, thus allowing microbial products deriving from gut microbiome to overcome mucosal surface and to reach lamina propria [7] and, subsequently, the hematic compartment [8]. This disruption of intestinal homeostasis allows the release of bacterial products in the circulation, or the so-called bacterial translocation, which is considered a major driver of chronic immune activation and disease progression in simian immunodeficiency virus (SIV)-infected rhesus macaques as well as in people living with HIV (PLWH) [9,10].

The natural history of HIV-1 infection has dramatically changed after the introduction of ART which allows for virologic control, thus halting disease progression [11,12]. However, people living with HIV (PLWH) still exhibit higher incidence of non-AIDS-related comorbidities and mortality compared to general population. Indeed, ART fails to completely extinguish the status of chronic immune activation and inflammation that persist in virologic-controlled, ART-receiving PLWH [13,14]. Such a population is consequently more prone to cardiovascular, liver, renal, bone dysfunction, as well as cardiovascular and neurocognitive diseases and cancer [15,16,17].

Several studies investigated the effect of ART to reconstitute the Th17 pool which is loudly reduced after HIV-1 infection. Today, it is accepted that ART is unable to reconstitute the Th17 depletion that seems to persist indefinitely in the course of the infection [5,18,19,20,21]. An exception is a study by Schuetz et al. documenting a positive outcome when ART is introduced in very early stages of acute HIV-1 infection [22]. On the other hand, just a limited number of reports have investigated the ART effect on local immune activation that is observed within the gut mucosa with as much contradictory results, discussed below [22,23,24,25].

With this in mind, we decided to address the effectiveness of ART in reversing the local (mucosal) CD4^+^ T lamina propria lymphocytes (LPLs) depletion and chronic immune activation observed during HIV-1 infection within the gut. To do this, we conducted a cross-sectional observational study to compare frequencies and immune activation status of IFN-γ producing (Th1) and Th17 CD4^+^ LPLs between treatment-naïve and long-term treated PLWH.

## 2. Materials and Methods

### 2.1. Study Partecipants

PLWH were recruited at the Division of Infectious Diseases, Hospital of Sapienza University of Rome (Italy) and subsequently divided into two study groups: ART-naïve PLWH and long-term ART-treated PLWH.

Inclusion criteria for the ART-naïve PLWH group were: age > 18 years, HIV-1 infection of new diagnosis, no previous ART treatment, and blood CD4^+^ cell count > 200/μL. Inclusion criteria for the long-term ART-treated PLWH group were to have: age > 18 years, HIV-1 infection, previous exposure to ART, HIV-1 viral load under the detection limit (37 copies/mL), and blood CD4^+^ cell count > 200/μL. Exclusion criteria for both PLWH study groups were: inflammatory bowel diseases, other gastroenterological diseases, HIV-related non-AIDS-defining comorbidities, and the presence of opportunistic infection.

A healthy control group, composed by people not living with HIV, was recruited at the Division of Gastroenterology of Sapienza University of Rome (Italy). It was composed of people who joined a colorectal cancer-screening program on the basis of familiarity for such disease and who were eligible for total colonoscopy and retrograde ileoscopy. Inclusion criteria included a negative result of the screening endoscopic procedure for any gastrointestinal disease. Exclusion criteria were HIV-1 infection, IBD, and/or other gastroenterological diseases.

The study was approved by the institutional review board (Department of Public Health and Infectious Diseases, University of Rome ‘‘Sapienza’’ and Ethics Committee Azienda Policlinico Umberto I of Rome). All study participants gave written informed consent.

Clinical anamnestic data were obtained from medical records.

### 2.2. Sample Collection

Twenty milliliters of whole blood were collected by venipuncture in Vacutainer tubes containing ethylene-diamine-tetra-acetic acid (EDTA) (BD Biosciences, San Jose, CA, USA). Plasma was immediately separated by centrifugation and stored at −80 °C for further analysis. All participants underwent a total colonoscopy and retrograde ileoscopy for at least 10 cm of distal ileum with conventional or slim scope (model CF or PCF-160 AI, Olympus Medical Europe GmbH, Hamburg, Germany). Two biopsies from five different intestinal sites (terminal ileum, cecum, ascending, transverse, and descending) were obtained, isolating LPLs from a total of 220 biopsies.

### 2.3. Specimen Processing

Briefly, gut biopsies from each patient were collected in RPMI 1640 (heat inactivated 10% fetal bovine serum), gathered together and washed twice with EDTA wash media, resuspended, and incubated for 1 h at room temperature in EDTA solution 5 mmol/L on an automatic shaker. Supernatant containing intraepithelial lymphocytes was removed and biopsies were digested by 1 h incubation at 37 °C in prewarmed RPMI 1640 with 1 mg/mL collagenase (Sigma-Aldrich, Milan, Italy) and 1.5 U DNAse I (Sigma-Aldrich), leading to isolation of LPLs, then filtered through a 70 μm cell strainer (Becton Dickinson, Franklin Lakes, NJ, USA), as previously described [26].

### 2.4. Cell Activation, Surface Marker and Intracellular Marker Staining

Phenotypes and activation markers were evaluated by Miltenyi Biotec flow cytometer-MACSQuant Analyzer on isolated LPLs by the following antihuman monoclonal antibodies: CD3-PerCP, CD4-APC-Vio770, CD8-FITC, CD38-APC, and HLA-DR-PE (Miltenyi Biotec, Bergisch Gladbach, Germany). Gating analysis and data were analyzed using MACSQuantify software 2.5 (Miltenyi Biotec). To evaluate intracellular IL-17 and IFN-γ production, LPLs were cultured accordingly to our established protocol [27]. The following antihuman mAbs were used: CD3-PerCP, CD4-APC-Vio770, CD8-FITC, IL-17-PE, and IFN-γ-APC. The frequencies of gut CD4^+^ T LPLs (CD3^+^ CD4^+^) were calculated as ratio of the gut CD4^+^ T LPLs to total LPLs (CD4^+^ LPLs/total LPLs).

### 2.5. Virological Analysisg

HIV-1 RNA copy numbers were evaluated in plasma samples collected from whole blood obtained in EDTA-containing tubes and stored at −80 °C. Combined with Siemens Healthcare’s nucleic acid extraction technology, a quantitative measurement of human immunodeficiency virus (HIV-1) RNA based on real-time polymerase chain reaction (PCR) technology (Versant kPCR by Siemens Healthcare Diagnostic Inc., Tarrytown, NY, USA) was performed. The detection limit was 37 copies/mL.

### 2.6. Statistical Analysis

Statistical analyses and graphs were performed using GraphPad Prism software, version 8.2.1 (GraphPad Software Inc., La Jolla, CA, USA). Differences between treatment-naïve and long-term treated PLWH were analyzed by the Mann–Whitney U test. The results were given as medians, ranges, and percentages. For all tests, a *p*-value below 0.05 was considered significant.

Since our healthy control group was composed by both male and female participants, we decided not to use it to perform statistical analysis, but only to show reference values for people without HIV-1 infection.

## 3. Results

### 3.1. Study Population

Overall, 22 male PLWH and 7 healthy controls were included in this study. The demographic and clinical characteristics of PLWH are summarized in Table 1. All enrolled PLWH were negative for cytomegalovirus or Epstein–Barr virus IgM, and had no hepatitis B virus or hepatitis C virus coinfection; no opportunistic infections were recorded.

PLWH were further divided into two study groups: a naïve to treatment PLWH group and a long-term treated PLWH group. The ART-naïve group included 11 males (median age 46 years); they had a median detectable plasma viral loads of 37,862 copies/mL at the enrollment (interquartile range: 7906 to 55,784 copies/mL) and a median blood CD4^+^ T cells count of 412 cells/µL (interquartile range: 277 to 621 cells/µL). By contrast, long-term ART-treated PLWH (median age 48 years) included PLWH who had been treated for a median of 11 years (interquartile range: 9 to 20.5 years); at enrollment their viral load in blood were below the detection limit of 37 copies/mL and their median CD4^+^ T-cells count was 773 cells/µL of blood (interquartile range: 708 to 1350 cells/µL).

The healthy control group was composed by seven people joining a colorectal cancer screening program on the basis of familiarity for such disease. They were four men and three women of age ranging between 40 and 55, who were not presenting any other known pathological condition. The screening result was negative for colorectal cancer for all study participants.

### 3.2. Differences in Intestinal Overall CD4^+^ LPLs, Th1 and Th17 LPLs between ART-Naïve and Long-Term ART-Treated PLWH

We first analyzed CD4^+^ T lymphocytes frequencies in LPLs of ART-naïve and long-term ART-treated PLWH. A statistically significant difference in the CD4^+^ LPLs frequencies was observed between the two groups (*p* < 0.001), with ART-naïve PLWH showing lower frequencies of CD4^+^ LPLs (median 11%; IQR 7.5–16.1) compared to long-term ART-treated PLWH (median 46.12%; IQR 42.4–51.2).

**Figure 1:**
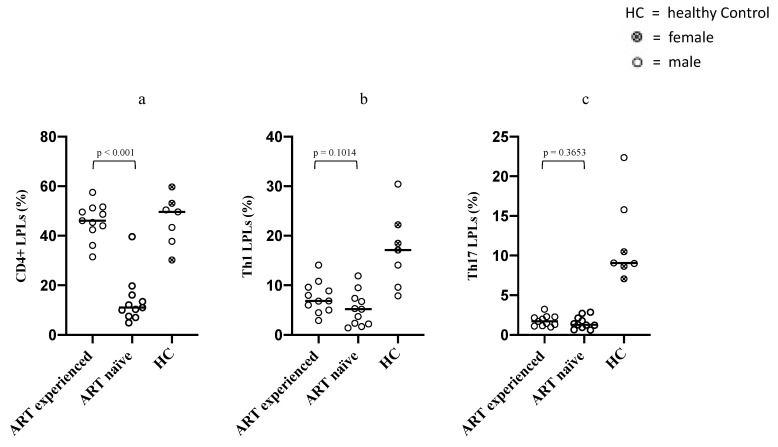
This figure shows comparison for overall CD4^+^ LPLs (**a**), IFN-γ expressing (Th1) CD4^+^ LPLs (**b**) and IL-17 expressing (Th17) CD4^+^ LPLs (**c**) detected within mucosal biopsies between ART-naïve PLWH and long-term ART-treated PLWH. Each dot represents a patient biopsy, while the horizontal line represents the median. The healthy control group’s results are shown on the right of each panel. *p*-values were determined by Mann–Whitney U test.

Then, we compared gut mucosal LPLs subsets between naïve and long-term treated PLWH by measuring the frequencies of CD4^+^ LPLs subsets, namely Th1 (IFN-γ expressing) and Th17 (IL-17 expressing) in LPLs collected from all participants. Interestingly, we found no statistically significant differences between the two groups [ART naive versus long-term ART-treated PLWH: Th1 (5.17% versus 6.84%) (*p* = 0.1); Th17 (1.29% vs. 1.72%) (*p* = 0.36)] (Figure 1).

### 3.3. Differences in the Immune Activation Status of Intestinal CD4^+^ LPLs between ART-Naïve and Long-Term ART-Treated PLWH

**Figure 2:**
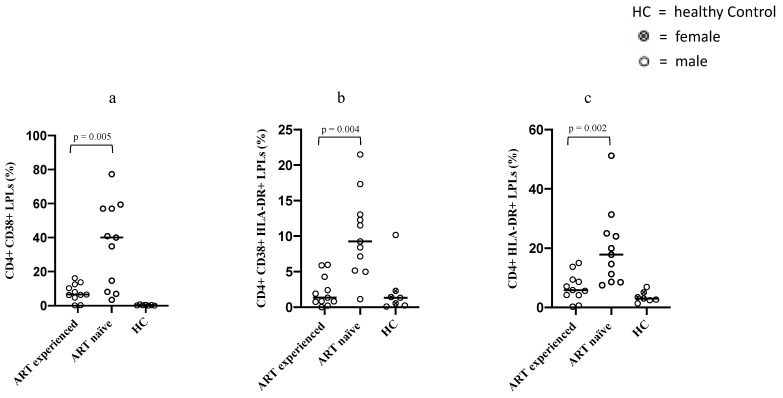
This figure shows comparison for CD38^+^ CD4^+^ LPLs (**a**), HLA-DR^+^ CD4^+^ LPLs (**b**) and double positive CD38^+^ HLA-DR^+^ CD4^+^ LPLs (**c**) detected within mucosal biopsies between ART-naïve PLWH and long-term ART-treated PLWH. Each dot represents a patient biopsy, while the horizontal line represents the median. The healthy control group’s results are shown on the right of each panel. *p*-values were determined by Mann–Whitney U test.

In order to assess whether the higher amount of CD4^+^ LPLs detected among long-term ART-treated PLWH was accompanied by a higher or lower degree of immune activation, we compared CD4^+^ LPLs immune activation by measuring the frequencies of expressing CD38 and/or HLA-DR CD4^+^ LPLs between the two groups. Our findings indicated that the median frequencies of CD4^+^ LPLs expressing CD38+ or HLA-DR^+^ were higher in the ART-naïve group compared to the long-term ART-treated PLWH group [CD4^+^ CD38^+^ LPLs: 40% versus 6.5% (*p* = 0.005); CD4^+^ HLA-DR^+^ LPLs: 17.8% versus 5.8% (*p* = 0.002)]. The same trend was observed for the median frequencies of CD4^+^ LPLs co-expressing both CD38 and HLA-DR surface markers [double positive CD4^+^ CD38^+^ HLA-DR^+^ LPLs: 9.2% versus 1.3% (*p* = 0.004)] (Figure 2).

## 4. Discussion

Intestinal mucosa is a preferential site of HIV-1 replication, associated with a severe CD4^+^ LPLs depletion and a loss of mucosal integrity; the disruption of intestinal architecture and homeostasis that rapidly occurs after HIV-1 infection is thus considered a hallmark of HIV immunopathogenesis, characterized by epithelial cell apoptosis and lymphocytes depletion, hence the mucosal barrier impaired functionality. As described above, among CD4^+^ LPLs, Th17 are preferentially depleted from the gut of PLWH and this loss is considered a major cause for microbial translocation, chronic immune activation, and inflammation in both treated and untreated PLWH [2].

An increasing body of literature has examined the effects of ART on gastrointestinal immune recovery. Although inconsistencies in study designs and patients selection, there is a consensus about the inability of ART in reconstituting the pre-infection immunological milieu, with particular reference to Th17 lymphocytes frequencies and functionality [20]. Our study results are consistent with such data, since no relevant difference was detected between ART-naïve and ART-experienced PLWH in terms of Th1 nor Th17 LPLs frequencies. Moreover, median values of both PLWH groups resulted way under the reference values of the healthy control group, thus confirming the lack of an ART-related positive effect on the reconstitution of Th1 and Th17. Nonetheless, when comparing long-term treated and untreated PLWH, we recorded a significant increase in the overall CD4^+^ LPLs solely within the long-term treated PLWH group, with overall CD4^+^ LPLs frequencies almost reaching the reference level of the healthy control group. The explanation for this unexpected result could rely on the high plasticity of the functional identity of T helper lymphocytes, which has been described in both health and diseases, especially within the gut [28]. Thus, the observed increase in total LPLs could be due to an increase in CD4^+^ LPLs subsets different from Th1 and Th17 such as Th2, Th9, Th22, Tfh, and Tregs. Moreover, since frequencies of LPLs coming from cytofluorimetric analysis are expressed as relative abundances, we cannot rule out the influence of a decrease in total CD8^+^ LPLs on the observed increase in total CD4^+^ LPLs. Unfortunately, our study design focused on Th1 and Th17 CD4^+^ LPLs subsets, so that it was impossible for us to identify the CD4^+^ LPLs subset which are responsible for the documented increase in the global intestinal CD4^+^ LPLs pool. However, such interesting results suggest a beneficial effect of therapy on the global intestinal CD4^+^ LPLs pool and encouraged us to check whether this even slight improvement was accompanied by a favorable or non-mucosal immune activation context.

A very limited number of studies, to the best of our knowledge, investigated the rate of local immune activation observable within the mucosal compartment during HIV-1 infection, as well as the extent to which ART can affect it. Yukl et al. compared immune activation status of LPLs between PLWH and healthy control, showing higher levels of CD38^+^ CD4^+^ LPLs but not HLD-DR^+^ LPLs among long-term (>12 months) ART-treated PLWH, but their study lacks an ART-naïve PLWH control group [23]. Similarly, Mehandru et al. longitudinally studied immune activation gastrointestinal levels of PLWH with acute and early infection providing a healthy control comparison group, detected CD4^+^ LPLs activation that was persistent over time during ART. They showed high levels of HLA-DR^+^ CD4^+^ LPLs up to 3 years after ART initiation, yet not in a subgroup of PLWH who were receiving ART for 3–7 years [24]. On the contrary, Sheth et al. found similar levels of immune activation, assessed by the expression of the surface marker HLA-DR, between long-term ART-treated PLWH and uninfected controls [25].

The latter study is in line with our current and previous [26] results, since we were able to observe significantly lower values of immune activation among long-term treated PLWH compared to ART-naïve controls. As a matter of fact, this pattern was consistent among all three LPLs subsets examined in our study, since both CD38^+^ and HLA-DR^+^ CD4^+^ LPLs, as well as double positive CD38^+^ HLA-DR^+^ CD4^+^ LPLs from long-term treated PLWH showed significantly lower frequencies than LPLs from ART-naïve PLWH.

The significant decrease in the observed lamina propria CD4+ LPLs immune activation is an important finding, especially in the context of mucosal dysfunction that is observed in HIV-1 infection immunopathogenesis. After HIV-1 infection has established and GALT depletion has started, focal breaches of the gastrointestinal mucosa allow the continuous stimulation of gut resident LPLs, which results in them being continuously exposed to antigens represented by microbial products enabled to cross the lamina propria layer [29]. Nonetheless, we observed a significant reduction in the mucosal immune activation status that was not affected by the lack of recovery in the Th17 subset, suggesting that other factors different from Th17 alone might play a relevant role driving immune activation in gut mucosal site.

It has been proposed that the failure in the recovery degree of Th17 loss despite ongoing ART might impair both the restoration of the mucosal barrier and the clearance of microbial products [30]. The quality, as well as the quantity, of such constant antigenic stimulation was called in play to account for the mucosal immune activation, with regard to the gut microbiome dysbiosis that occurs during HIV-1 infection. Copious evidence has emerged on the modulating properties of probiotics in the context of HIV-related immune activation, as confirmed by our previous works [31,32]. Furthermore, associations between mucosal cellular immune activation and mucosa-adherent bacteria (such as an increase in the pathobiont *Prevotella* abundance [33], as well as a decrease in the butyrate-producing *Roseburia* abundance [34]) have been described. With all this in mind, the reduced immune activation that we observed only among long-term ART-treated PLWH could be considered the proxy of a quantitative reduction in the amount of the microbiologic stimuli able to reach the mucosal compartment, thus suggesting a beneficial effect of ART on the recovery of gastrointestinal mucosa barrier competence. Otherwise, changes in the qualitative microbiome composition, induced by a yet unknown microflora modulating ART effect, could account for the reduced immune activation, as described.

The current study presents some limitations. First, our sample size is small and our PLWH cohorts solely include men, which may lead to bias occurrence. Second, due to the limited number of LPLs obtainable from gut biopsies, we were forced to gather together LPLs, so we were not able to compare different gut sites biopsies in terms of LPLs reconstitution and immune activation. Third, our healthy control group was small and included both male and female, thus impeding a proper comparison among groups. Finally, the lack of data deriving from blood and stool did not allow us to determine whether the reduced immune activation observed in the intestinal mucosa of long-term treated PLWH is accompanied by a reduction in peripheral immune activation or modulation of gut microbiome. Still, being the intestinal mucosa at midway between gut microbiome and the hematic compartment, such a reduction in mucosal immune activation is intriguing and warrant further studies to highlight the link between the observed immunological changes, gut microbiome, and health.

## 5. Conclusions

In conclusion, our study showed that, despite ART failing to restore the Th1 and Th17 levels within the gut mucosa, it is effective in increasing overall CD4^+^ T LPLs frequencies and reducing mucosal immune activation. In the future, more comprehensive studies are warranted to understand the complex relationship between HIV-1-associated gut microbiome dysbiosis, lamina propria immune properties, and ART-related modulating effects.

## Figures and Tables

**Table 1:** Age, immune and virologic profile of study population.

	ART-Naïve (*n* = 11)	ART Experienced (*n* = 11)
parameter (unit)	median (IQR ^1^)	median (IQR ^1^)
Age (years)	46 (39–49)	48 (32–56)
Viral load at enrollment (copies/mL)	37,862 (7906–55,784)	<37
Blood CD4^+^ T cells count at enrollment (cells/µL)	412 (277–612)	773 (708–1350)
Nadir ^2^ blood CD4^+^ T cells (cells/µL)	363 (310–505)	360 (337–485)
ART exposure time (years)	-	11 (9–20)

^1^ IQR: Interquartile range (25–75%); ^2^ The nadir of blood CD4^+^ T cells is the lowest value of blood CD4^+^ T cells ever recorder along the medical history of PLWH.

## Data Availability

No applicable.
